# Horses for courses? Assessing the potential value of a surrogate, point‐of‐care test for SARS‐CoV‐2 epidemic control

**DOI:** 10.1111/irv.12796

**Published:** 2020-08-06

**Authors:** Sharif A. Ismail, Catherine Huntley, Nathan Post, Samuel Rigby, Madhumita Shrotri, Sarah V. Williams, Sharon J. Peacock

**Affiliations:** ^1^ Department of Global Health and Development London School of Hygiene and Tropical Medicine London UK; ^2^ Department of Primary Care and Public Health Imperial College London London UK; ^3^ Faculty of Public Health and Policy London School of Hygiene and Tropical Medicine London UK; ^4^ Department of Medicine University of Cambridge Cambridge UK

**Keywords:** antibody, COVID‐19, diagnostic, molecular, rapid diagnostic test, SARS‐CoV‐2

## Abstract

Point‐of‐care tests (POCTs) offer considerable potential for improving clinical and public health management of COVID‐19 by providing timely information to guide decision‐making, but data on real‐world performance are in short supply. Besides SARS‐CoV‐2‐specific tests, there is growing interest in the role of surrogate (non‐specific) tests such as FebriDx, a biochemical POCT which can be used to distinguish viral from bacterial infection in patients with influenza‐like illnesses. This short report assesses what is currently known about FebriDx performance across settings and populations by comparison with some of the more intensively evaluated SARS‐CoV‐2‐specific POCTs. While FebriDx shows some potential in supporting triage for early‐stage infection in acute care settings, this is dependent on SARS‐CoV‐2 being the most likely cause for influenza‐like illnesses, with reduction in discriminatory power when COVID‐19 case numbers are low, and when co‐circulating viral respiratory infections become more prevalent during the autumn and winter. Too little is currently known about its performance in primary care and the community to support use in these contexts, and further evaluation is needed. Reliable SARS CoV2‐specific POCTs—when they become available—are likely to rapidly overtake surrogates as the preferred option given the greater specificity they provide.

## 1 BACKGROUND

The COVID‐19 pandemic presents health policymakers and clinicians with difficult decisions under conditions of uncertainty, facing an infection that in its early stages mimics many other influenza‐like illnesses (ILIs).^1^ The range of diagnostic tests available for SARS‐CoV‐2 is growing, but they have important limitations. RT‐PCR is the reference standard but depends on advanced laboratory facilities that are not available in primary care or community settings, and long turn‐around times for results increase the risk of nosocomial transmission.^2^ Performance profiles for RT‐PCR tests for SARS‐CoV‐2 are also sub‐optimal, with reported false‐negative rates of up to 30%.^3^

Point‐of‐care tests (POCTs) could transform epidemic control by better guiding triage decisions to reduce risks of nosocomial and community transmission. However, reliable POCT options in the period before a detectable antibody response has been mounted are few. There could therefore be a role for surrogate tests (those that do not directly diagnose SARS‐CoV‐2) for screening. These include FebriDx, a low‐cost POCT used to distinguish bacterial from viral infections and originally designed to support rational antibiotic prescribing.^4^ A number of evaluations are ongoing to consider the value of FebriDx in acute and primary care settings during the COVID‐19 pandemic.^5^, ^6^

This short report explores the place of surrogate POCTs in the diagnostic mix in high‐income settings, by considering the performance of FebriDx and SARS‐CoV‐2‐specific POCTs on the market.

## 2 POCT MODALITIES AND SARS‐CoV‐2 IN THEORY

There are four POC testing approaches for SARS‐CoV‐2 with different decision‐making implications:

- Class I: POCTs for the presence of SARS‐CoV‐2 antigens to identify active infection.^7^
- Class II: molecular POCTs to identify active infection but with shorter turn‐around times than conventional RT‐PCR.
- Class III: serological antibody tests (IgG and/or IgM) for confirmation of infection. This includes most SARS‐CoV‐2‐specific POCTs.
- Class IV: surrogate tests (including FebriDx) that do not directly diagnose SARS‐CoV‐2 but can be used as screening tests.

Point‐of‐care test utility varies according to time from symptom onset. PCR‐based tests offer better sensitivity in the first few days, but performance declines from as early as day 5.^8^ Class III POCTs likely offer value only from day 7 to 8 because of the time taken to generate a detectable antibody response.^9^, ^10^, ^11^ All POCTs are, for now, used to guide interim decision‐making pending final diagnosis via RT‐PCR.

While class I, II and III modalities test for SARS‐CoV‐2‐specific antigens, RNA or antibodies respectively, FebriDx is a composite tool that detects both myxovirus resistance protein A (MxA—elevated in acute viral infections) and C‐reactive protein (CRP—elevated in either viral or bacterial infections). The test uses fixed thresholds for “positive” MxA and CRP levels; any positive MxA result, with or without positive CRP, indicates viral infection.^4^ These markers are used because: (a) CRP production is stimulated by interleukin‐6 (IL‐6), produced in higher quantities in bacterial infections; (b) MxA expression is exclusively driven by type 1 interferons (IFN‐1), secreted in response to detection of viral signatures by host intracellular receptors. IFN‐1 and consequently MxA are thought to be specific to viral infection.^4^

There may be grounds for caution in interpreting FebriDx results at the extremes of the clinical course. Early SARS‐CoV infection appears not to activate MxA and IFN‐1 transcription in the normal way,^12^ and if SARS‐CoV‐2 similarly attenuates early MxA expression, FebriDx may have low sensitivity in at least the first few days following infection. The low MxA threshold for a positive result (40 ng/mL) means FebriDx may nevertheless be positive even with weak MxA activation.

## 3 COMPARATIVE PERFORMANCE OF SURROGATE AND SPECIFIC TESTS FOR SARS‐CoV‐2

The key risk in using any POCT is of a false‐negative result leading to inappropriate management of SARS‐CoV‐2 infection. For FebriDx, an additional risk is that a positive result cannot exclude the possibility of another virus as the cause of infection.

Studies evaluating FebriDx mostly consider performance in distinguishing viral from bacterial causes of acute respiratory illnesses in toto, in secondary care. Reported sensitivities range from 64% to 90%, and specificities from 78% to 88%.^13^, ^14^, ^15^, ^16^ However, two recent studies from the UK evaluate FebriDx specifically for screening for SARS‐CoV‐2 in hospital: a small‐scale pilot,^5^ and a study nested within a non‐randomised clinical trial of molecular POCTs.^6^ These studies report impressive sensitivities and specificities of 100% and 93%, and 100% and 86%, respectively. However, interpretation is limited by (a) the use of clinical diagnosis as reference standard rather than RT‐PCR in the first study, (b) evaluation in single secondary care centres in England in both cases and (c) the inclusion of patients in the range 2‐7 days from symptom onset only in the first study. Finally, test performance in both studies may have been artificially boosted because they were conducted at times when the range of co‐circulating respiratory viruses was lower than in the autumn and winter.

**FIGURE 1:**
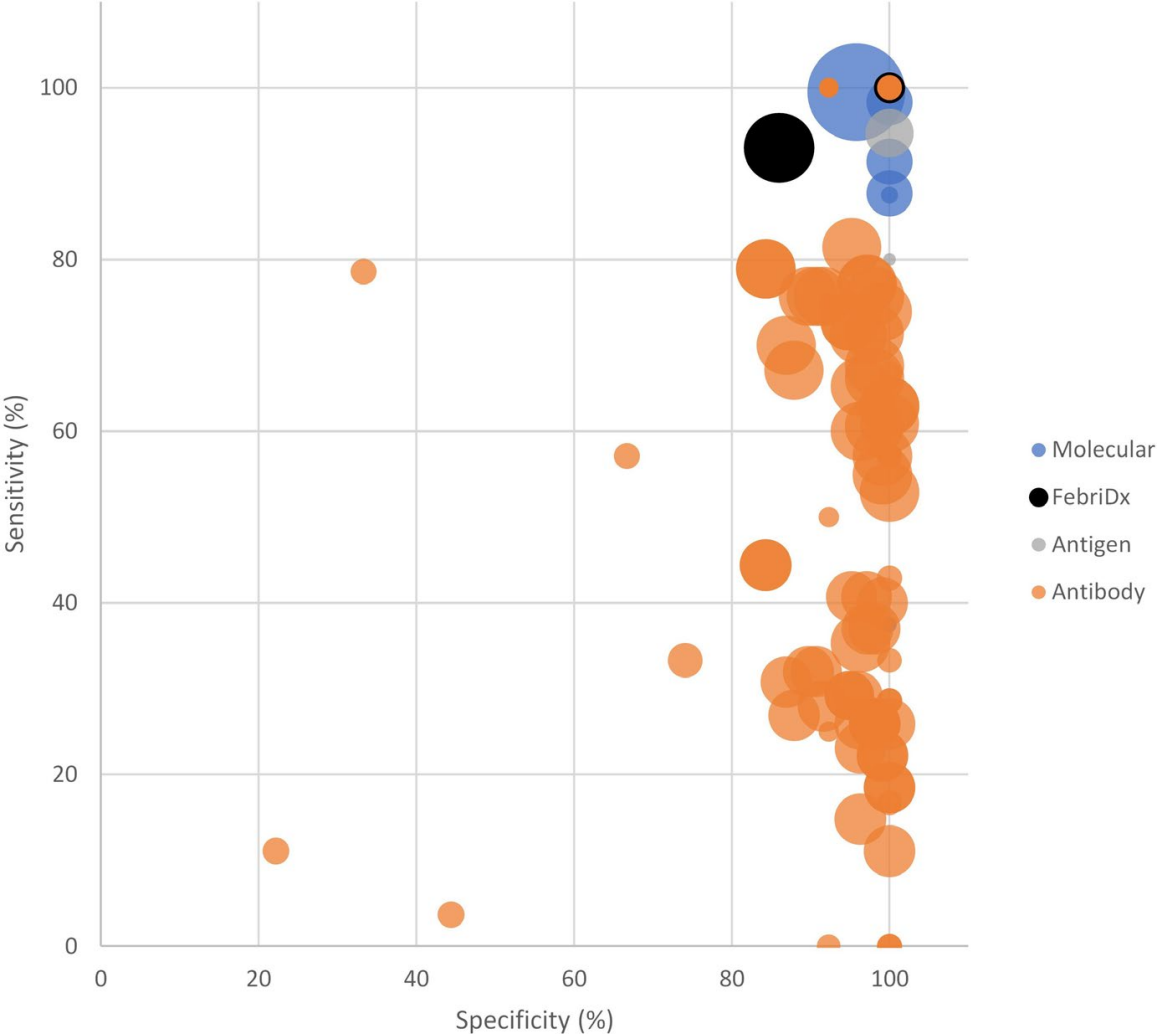
Scatter plot of sensitivity and specificity values for antigen‐ and antibody‐based POCTs, molecular rapid diagnostic tests and scores for FebriDx from the two studies that specifically evaluate performance in screening for COVID‐19 published thus far. Bubble sizes indicate the size of the population involved in each study; the centre point of each bubble gives the sensitivity and specificity point estimate [Source: FIND Dx open‐source data on test performance^17^

A comprehensive assessment of POCT field performance is beyond the scope of this paper, and available data indicate large context‐dependent variations even for the same platform. However, based on data covering the first 14 days from symptom onset collated by FIND,^17^ SARS CoV2‐specific tests perform comparably to FebriDx (Figure 1). Caveats to this assessment are that: (a) the majority of tests are antibody‐based and therefore only reactive some time after symptom onset, and (b) most studies used patient samples collected in clinical settings only. Evidence on the performance of FebriDx or any other POCT for diagnosis of SARS‐CoV‐2 infection in key workers, care home residents or other high‐risk populations is in very short supply.

## 4 IMPLICATIONS FOR APPLICATIONS OF FEBRIDX

What can be said about the utility of FebriDx by comparison with other POCTs given the limited available data? Preliminary judgements are possible depending on the (a) timing and location of testing, and (b) the broader epidemiological context.

FebriDx offers greatest value early in the clinical course, given the absence of reliable antigen POCTs and scarcity of molecular POCTs. However, reported sensitivity ranges for COVID and non‐COVID patients in healthcare settings are wide.^5^, ^6^, ^13^, ^14^, ^15^, ^16^ The test should therefore be positioned within a diagnostic algorithm, alongside other clinical and radiological markers, to optimise performance. FebriDx may have a role in triage in the community where access to advanced diagnostics is limited, but there are no performance data for the test in this context, and only a handful of studies evaluate any SARS‐CoV‐2 POCTs in the community.^2^

Secondly, surrogate tests such as FebriDx will likely only have value around the peak of the epidemic curve, in a situation where widespread community transmission is probable. Here, a positive result in a patient with an ILI could be interpreted as indicative of COVID‐19 pending laboratory confirmation. Discriminatory power will likely decline during the autumn and winter months where other viral diagnoses are equally or more likely. Here, the clinical and social cost of imposing infection control measures such as isolation on the basis of a presumptive diagnosis alone may become unjustifiable.

## 5 CONCLUSION

Although a growing number of specific and surrogate POCTs are now available for SARS‐CoV‐2, test performance is variable across the clinical course. Given existing data, its ease of use and low cost, FebriDx shows promise as a screening tool for early‐stage COVID‐19 infection in hospital settings. However, not enough is yet known about its value in primary care or community settings. Reliable, SARS CoV2‐specific tests—when they become available—are likely to be the preferred option especially during the autumn and winter months when the incidence of other, co‐circulating respiratory viral infections will affect the discriminating power of surrogate tests like FebriDx.

## CONFLICT OF INTEREST

All authors declare that they have no conflicts of interest in relation to this work.

## AUTHOR CONTRIBUTIONS

**Sharif A. Ismail:** Conceptualization (equal); investigation (equal); writing – original draft (lead); writing – review and editing (equal). **Catherine Huntley:** Investigation (equal); writing – review and editing (equal). **Nathan Post:** Investigation (equal); writing – review and editing (equal). **Samuel Rigby:** Investigation (equal); writing – review and editing (equal). **Madhumita Shrotri:** Investigation (equal); writing – review and editing (equal). **Sarah V. Williams:** Investigation (equal); writing – review and editing (equal). **Sharon J. Peacock:** Conceptualization (equal); writing – review and editing (equal).

## References

[1] Zhou F , Yu T , Du R , et al. Clinical course and risk factors for mortality of adult inpatients with COVID‐19 in Wuhan, China: a retrospective cohort study. Lancet. 2020;395(10229):1054‐1062. 32171076 10.1016/S0140-6736(20)30566-3 PMC7270627

[2] Döhla M , Boesecke C , Schulte B , et al. Rapid point‐of‐care testing for SARS‐CoV‐2 in a community screening setting shows low sensitivity. Public Health. 2020;182:170‐172. 32334183 10.1016/j.puhe.2020.04.009 PMC7165286

[3] Arevalo‐Rodriguez I , Buitrago‐Garcia D , Simancas‐Racines D , et al. False‐negative results of initial RT‐PCR assays for COVID‐19: a systematic review. medRxiv. 2020 (ref 2020.04.16.20066787). 10.1371/journal.pone.0242958 PMC7728293 33301459

[4] NICE . FebriDx for C‐reactive protein and Myxovirus virus resistance protein A testing in primary care: A testing in primary care Medtech innovation briefing. London, UK: NICE; 2017.

[5] Karim N , Ashraf MZ , Naeem M , et al. Utility of FebriDx in early identification of possible COVID19 infection. Res Sq. 2020;1‐12. 10.21203/rs.3.rs-25802/v1

[6] Clark TW , Brendish NJ , Poole S , et al. Diagnostic accuracy of the FebriDx host response point‐of‐care test in patients hospitalised with suspected COVID‐19. J Infect. 2020. S0163‐4453(20)30432‐1. 10.1016/j.jinf.2020.06.051 PMC7306108 32579983

[7] Cheng MP , Papenburg J , Desjardins M , et al. Diagnostic testing for severe acute respiratory syndrome‐related coronavirus‐2: a narrative review. Ann Intern Med. 2020;13:13. 10.7326/M20-1301 PMC7170415 32282894

[8] Wölfel R , Corman VM , Guggemos W , et al. Virological assessment of hospitalized patients with COVID‐2019. Nature. 2020;581:465‐469. 32235945 10.1038/s41586-020-2196-x

[9] Zhao J , Yuan Q , Wang H , et al. Antibody responses to SARS‐CoV‐2 in patients of novel coronavirus disease 2019. Clin Infect Dis. 2020;28. ciaa344. 10.1093/cid/ciaa344 PMC7184337 32221519

[10] Guo L , Ren L , Yang S , et al. Profiling early humoral response to diagnose novel coronavirus disease (COVID‐19). Clin Infect Dis. 2020;71(15):778‐785. 32198501 10.1093/cid/ciaa310 PMC7184472

[11] Huang AT , Garcia‐Carreras B , Hitchings MDT , et al. A systematic review of antibody mediated immunity to coronaviruses: antibody kinetics, correlates of protection, and association of antibody responses with severity of disease. medRxiv. 2020 (ref 2020.04.14.20065771). 10.1038/s41467-020-18450-4 PMC7499300 32943637

[12] Spiegel M , Weber F . Inhibition of cytokine gene expression and induction of chemokine genes in non‐lymphatic cells infected with SARS coronavirus. Virol J. 2006;29(3):17. 10.1186/1743-422X-3-17 PMC1444920 16571117

[13] Beard K , Chan C , Mills S , Poole S , Brendish N , Tristan WC . Evaluation of the febridx host response point‐of‐care test to differentiate viral from bacterial etiology in adults hospitalized with acute respiratory illness during influenza season. Open Forum Infect Dis. 2019;6(Suppl 2):2019.

[14] Sambursky R , Shapiro N . Evaluation of a combined MxA and CRP point‐of‐care immunoassay to identify viral and/or bacterial immune response in patients with acute febrile respiratory infection. Eur Clin Respir J. 2015;2(1):28245. 26672961 10.3402/ecrj.v2.28245 PMC4676840

[15] Self W , Rosen J , Sharp S , et al. Diagnostic accuracy of FebriDx: a rapid test to detect immune responses to viral and bacterial upper respiratory infections. J Clin Med. 2017;6(10):94. 10.3390/jcm6100094 PMC5664009 28991170

[16] Shapiro NI , Self WH , Rosen J , et al. A prospective, multi‐centre US clinical trial to determine accuracy of FebriDx point‐of‐care testing for acute upper respiratory infections with and without a confirmed fever. Ann Med. 2018;50(5):420‐429. 29775092 10.1080/07853890.2018.1474002

[17] FIND . SARS‐CoV2 diagnostics: performance data. 2020. https://www.finddx.org/covid‐19/dx‐data/. Accessed June 02, 2020.

